# Gestational diabetes is associated with alteration on pelvic floor muscle activation pattern during pregnancy and postpartum: Prospective cohort using electromyography assessment

**DOI:** 10.3389/fendo.2022.958909

**Published:** 2022-10-06

**Authors:** Caroline Baldini Prudencio, Sthefanie Kenickel Nunes, Fabiane Affonso Pinheiro, Carlos Izaias Sartorão Filho, Guilherme Thomaz de Aquino Nava, Sauro Emerick Salomoni, Cristiane Rodrigues Pedroni, Marilza Vieira Cunha Rudge, Angélica Mércia Pascon Barbosa, Diamater Study Group, M. V. C. Rudge

**Affiliations:** ^1^ São Paulo State University (Unesp), Postgraduate Program on Tocogynecology, Botucatu Medical School, Botucatu, Brazil; ^2^ Human Development and Technologies, Institute of Biosciences, São Paulo State University (UNESP), Rio Claro, Brazil; ^3^ Independent Researcher, Hobart, TAS, Australia; ^4^ São Paulo State University (Unesp), School of Philosophy and Sciences, Marilia, Brazil

**Keywords:** gestational diabetes, pelvic floor, pregnant, electromyography, postpartum

## Abstract

**Background and objective:**

Gestational diabetes mellitus (GDM) is a comorbidity which may cause acute and lifelong disorders to mother and child. Alterations in muscular and connective tissues have been associated with GDM in translation studies, characterizing gestational diabetic myopathy. Pregnancy-specific urinary incontinence and sexual disabilities, disorders that depend on the pelvic floor muscle (PFM) integrity, are also associated with GDM both during and after pregnancy. The aim was to compare PFM activation patterns between GDM and non-GDM women from 24–30 gestational weeks to 18–24 months postpartum during a standard clinical test during gestation and postpartum.

**Methods:**

We conducted a prospective three-time-point cohort study from gestation (24–30 weeks—T1, and 36–38 weeks—T2) to 18–24 months postpartum (T3). PFM electromyography was recorded in primigravida or primiparous women with one previous elective c-section with or without the diagnosis of GDM according to the American Diabetes Association criteria. A careful explanation of the muscle anatomy and functionality of the PFM was given to participants before EMG assessment. The outcome measures were PFM activation patterns assessed during pregnancy and postpartum, comparing intra and between groups. PFM activation patterns were assessed by normalized electromyography signal at rest and during 1-second (sec) phasic, 10-sec hold, and 60-sec sustained contractions.

**Results:**

Demographic and obstetric data showed homogeneity between groups. The GDM group achieved peak PFM EMG amplitudes similarly to the non-GDM group, but they took longer to return to baseline levels during the ~1-sec contraction (flicks). During 10-sec hold contractions, the GDM group sustained lower levels of PFM activation than the non-GDM group at both 36–38 weeks of gestation and 18–24 months postpartum when compared to the non-GDM group.

**Conclusion:**

The results suggest that GDM impaired PFM control mainly on 1-sec flicks and 10-sec hold contraction, which appears to develop during late pregnancy and extends long-term postpartum. This motor behavior may play a role on pelvic floor dysfunctions.

## Introduction

Gestational diabetes mellitus (GDM) and gestational diabetic myopathy have been described as risk factors to pelvic floor muscle dysfunction (PFMD) during pregnancy and postpartum (1–10). Compromised PFM integrity may predispose women to PFMD such as pregnancy-specific urinary incontinence (PS-UI) (2) and postpartum urinary incontinence, which have substantial social and economic burden, in addition to high public health costs (11). More specifically, GDM has been associated with higher prevalence of both PS-UI and IU postpartum, with worsening of severity and quality of life during pregnancy and over the first year postpartum compared to non-GDM women (1–3, 5, 12). Taken together, current evidence indicates that PFM could be failing to perform contractions properly in women with GDM. A clinical triad composed of pelvic floor muscle (PFM) myopathy, PS-UI, and GDM is the focus of research. However, there is a lack of studies with longitudinal design assessing PFM function during and after pregnancy, especially in the GDM group (13).

Experimental studies in moderate diabetic rat models have shown that the periurethral and rectus abdominis muscles present deterioration, such as atrophy, thinning, disorganization, and co-localization of fast and slow fibers (7, 8, 10, 14). These data are consistent with those observed in rectus abdominis muscle tissues collected from pregnant women with GDM during C-section (6, 15), which suggests that GDM is indeed capable of damaging the muscular tissue causing a myopathic process (6, 15–17). Establishing a rational line by the morphological findings from urethral and rectus abdominis muscle of rats (7, 8, 10, 14) and rectus abdominis on pregnant women (6, 15), the PFM is also potentially impacted by the myopathic process (6). Due to the invasive nature of PFM biopsy, functional tests have been employed to evaluate the impact of GDM on its function. Electrophysiological tools (18–21) such as electromyography (EMG) have been used to understand PFM motor behavior during pregnancy, but fewer showed how GDM implies PFM function impairments when compared to non-diabetic pregnant women. In a study using electromyography (EMG), the amplitude of PFM signals during rest and hold contraction was decreased from the second to the third trimester. When three-dimensional ultrasonography (3D-US) was used, negative biometric changes, such as a low increase in the hiatal area, a decrease in the anteroposterior diameter, and a reduced levator ani muscle thickness, have also been observed between these two time points (16, 17).

Although previous studies have demonstrated impairments in PFM function associated with GDM, current evidence is still inconclusive in relation to the time frame in which these impairments evolve and whether women with GDM are capable of recovering PFM function after delivery (22). These are important clinical questions to understand the underlying pathophysiology of PFMD. Hence, the aim of this longitudinal study was to compare PFM activation patterns between GDM and non-GDM women from 24–30 gestational weeks to 18–24 months postpartum during a standard clinical test during gestation and postpartum.

## Methods

### Study design, participants, and group composition

This prospective cohort study was conducted in accordance with the Declaration of Helsinki and was approved by the Institutional Ethical Committee of the Botucatu Medical School of Sao Paulo State University (Protocol Number CAAE 82225617.0.0000.5411). The STROBE checklist was applied on the study. Written informed consent was obtained from all participants after careful explanation of all research procedures.

The inclusion criteria were as follows: pregnant women between 24 and 30 weeks of gestation in the first assessment; singleton pregnancy; 18–40 years of age; ability to perform a palpable contraction of the PFM (23); had not received PFM training or any musculoskeletal PFM treatment previously or during pregnancy. The exclusion criteria were clinical diagnosis of diabetes (type I or II, or overt diabetes in previous pregnancy), history of urinary incontinence (UI), having had more than two pregnancies, previous vaginal delivery, previous prolapse or incontinence surgery, failure to understand or follow the command to contract PFM, history of neurological diseases, visible genital prolapse, cervical isthmus incompetence, smoking, preterm birth, abortion, and participants who withdrew their consent during cohort.

The diagnosis guidelines proposed by the American Diabetes Association were used to identify patients with GDM (24) using the 75-g oral glycemic tolerance test (75g-OGTT). The test was applied to all participants at 24 gestational weeks, and participants were assigned to the GDM group if they presented fasting glycemic levels ≥92 mg/dl or 1 h ≥180 mg/dl or 2 h ≥153 mg/dl. Conversely, participants who had lower glycemic levels were allocated to the non-GDM group.

### Participant recruitment and assessment

Participants were evaluated at three time points: 24–30 weeks of gestation (T1), at 36–38 weeks of gestation (T2), and 18–24 months postpartum (T3). The same procedures were followed at each time point.

Eighty-two participants between 24 and 30 weeks of gestation who met the criteria were recruited from the Perinatal Diabetes Research Center (PDRC) of Botucatu Medical School/UNESP/Brazil, between 2017 and 2019. After giving their written consent, they were invited to answer a questionnaire with personal details; clinical and obstetric historic and anthropometric measures were taken.

Afterward, the PFM examination was explained and subsequently conducted by a single trained physiotherapist (CBP) with 4 years of experience in PFM evaluation. After emptying their bladder, participants were asked to lie down on the stretcher in supine position with their lower limbs flexed. Explanation about the anatomy and function of PFM was provided. To guarantee that participants understood the instructions, vaginal digital palpation was performed, and a PFM contraction was requested by giving the verbal instruction “squeeze the vaginal muscle and hold them as hard as possible, as if you were holding urine until I say to relax”. Visual inspection was held to ensure an isolated PFM contraction was well executed, without unusual/excessive co-contraction of the adductor and gluteus, hip movements, or expulsion movements (16, 25, 26). Afterward, participants were asked to perform a short sequence of PFM contractions, in preparation to the Glazer protocol of clinical evaluation (27) that would be used for the PFM EMG assessment: three brief contractions of 1-sec (Flick) phasic contraction and three contractions sustained for 10-sec (Hold) sustained PFM contractions. Participants received strong verbal encouragement and during contractions, and digital palpation was used to confirm that they performed maximal voluntary contractions (MVCs) on every attempt. During the 5-min rest period before EMG recordings, additional instructions were given depending on the performance, any possible doubts were clarified addressed, and the instruction to contract the PFM as hard as they could before relaxing was reinforced.

### EMG recordings and experimental protocol

**Figure 1:**
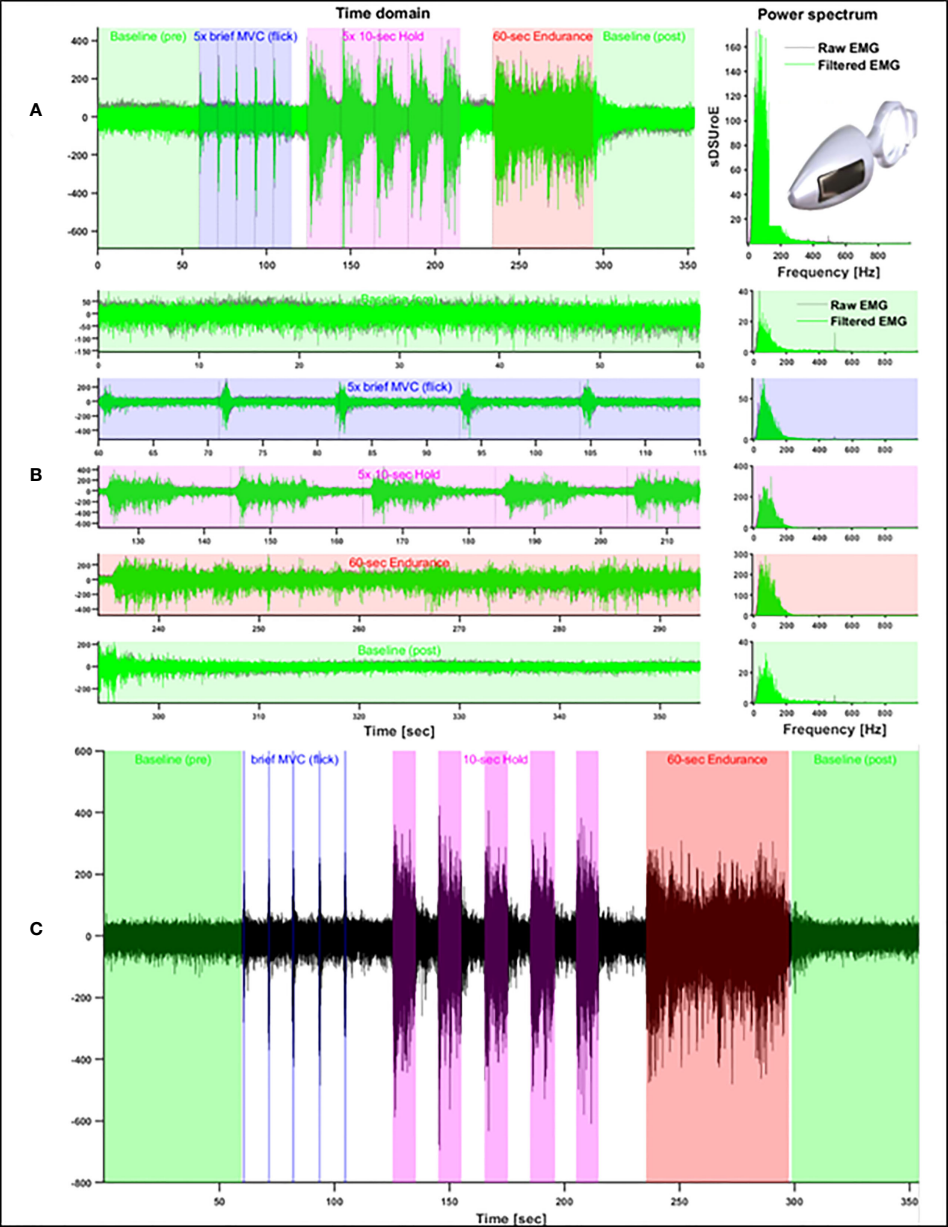
Glazer Protocol plots showing full signal and intravaginal probe image **(A)**, protocol segment tasks **(B)** and contraction time window with all performed tasks **(C)**.

The EMG signals were recorded using a two-channel device (Miotool 200 Uro; Porto Alegre, Brazil) with a gain of 1,000, a 14-bit A/D converter, an input impedance of 10 (10) Ohm/2 pF, and a common mode rejection ratio (CMRR) at 126 dB. Signals were sampled at 2,000 Hz. PFM EMG was recorded using only one channel and an intravaginal probe sensor (**Figure 1A**) with two opposite stainless-steel electrodes (85 × 25 mm) positioned on both sides of the vaginal sidewall, coupled to a differential sensor with a ring connection. A water-soluble gel was applied before introducing the probe into the vaginal canal. The reference electrode was placed on the ulna’s styloid process following the SENIAM recommendations (28).

The Glazer clinical protocol (**Figure 1A**) was used to standardize PFM activation. The protocol consists of the following sequence: (i) a 60-sec rest (Baseline-pre); (ii) brief 1-sec phasic contractions (Flicks) repeated five times, followed by a 10-sec rest interval; (iii) 10-sec sustained contractions (Hold) repeated five times, with a 10-sec rest in between; (iv) a 60-sec sustained endurance contraction (Endurance); and (v) a 60-sec rest (Baseline-post) (27, 29) (**Figure 1B**). The following verbal instructions were given to all participants to explain the execution of each task: (i) “Please, stay relaxed as quite as possible, until I say to you to contract the PFM” (Baseline-pre); (ii) “Please, squeeze your vaginal and anus muscles as harder as possible and relax as soon as instruct you” (Flick); (iii) “Please, squeeze and hold your vagina and anus as harder and as long as possible until 10 seconds” (Hold). They were encouraged to sustain the MVC during 10 sec by the verbal instruction: “keep squeezing, keep going, keep going”; (iv) during the 60-sec endurance contraction, the same instruction used for the 10-sec Hold contraction was given, but the instruction to “keep squeezing, contract, keep going, contract as harder as possible, keep going” was continuously repeated during the 60 sec; (v) and in the last 60-sec rest period (baseline-post), participants were instructed to “Relax your vagina and anus as much as possible and stay relaxed for 60-sec”.

### Electromyographic signal processing

The EMG signals were processed offline using custom programs using a band-pass filter of 20–500 Hz implemented in MATLAB (2014b, The MathWorks, Inc., Natick, MA, USA). First, the quality of the signals from each data collection was evaluated based on visual inspection and signal-to-noise ratio (SNR). Recordings with a low SNR, where the EMG was not discernible from the background or contains excessive signal artifacts, were removed from the analyses (n = 13). Because we detected significant contamination from the power line (60 Hz), an adaptive least mean squares (LMS) filter was implemented, using MATLAB function dsp.LMSFilter, in order to selectively remove contamination at 60 Hz and higher harmonics. The central frequency of the filter was adjusted in each case, depending on the presence of contamination in each harmonic.

The EMG profiles were obtained by applying the root mean square (RMS) function to the entire signal using a sliding window of 200 msec. Consistent with previous studies using the same protocol, the RMS EMG profiles were then normalized by the highest peak detected across the five repetitions of the Flick task (30). Although the Glazer protocol defines fixed time windows for the execution of each task, we ensured the precise selection of time windows of each contraction task by using a single-threshold algorithm to automatically detect the EMG onset and offset of muscle activity (31), which were confirmed by visual inspection (see **Figure 1**). Rest periods (Baseline-pre and -post) were initially selected from the timing expected from the protocol and were also visually inspected, with adjustments when necessary.

**Figure 2:**
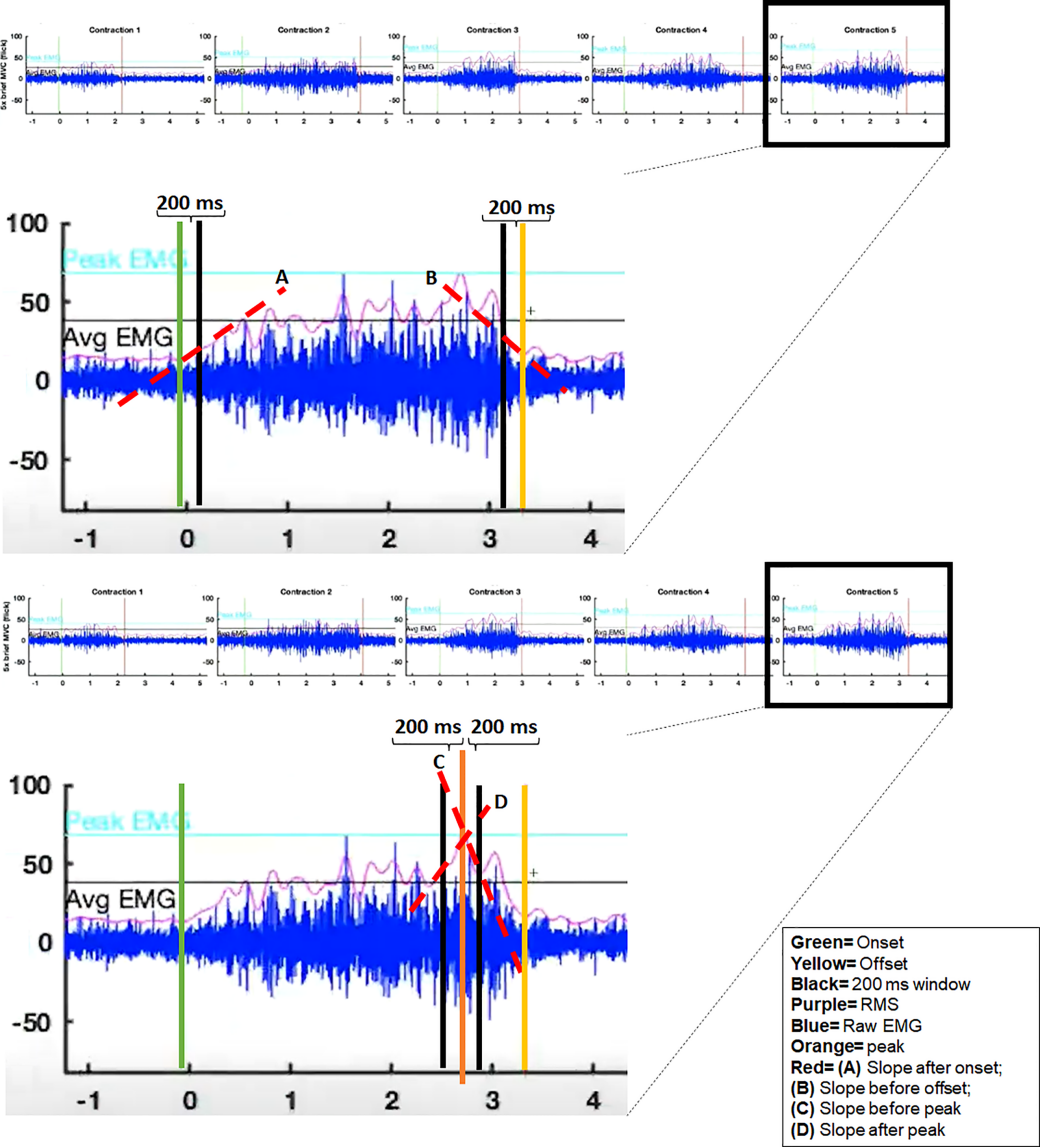
Example EMG recording of the Flick task from a representative subject, illustrating the EMG variables used in the analyses.

To characterize the muscle activation patterns of each subject, we extracted the following parameters from the normalized RMS EMG profiles of each task: average and peak amplitudes, standard deviation of the amplitude, and coefficient of variation. For the Flick and Hold tasks, we also extracted the time from EMG onset to peak amplitude and the time from peak amplitude to EMG offset. Using time windows of 200 msec, we also estimated the slopes (%/sec) of the RMS EMG after EMG onset (i.e., “increase rate of activity”) and before EMG offset (i.e., “decrease rate of activity”), as well as the slopes before and after the time of peak amplitude (**Figure 2**).

Finally, the full RMS EMG waveforms from the Flick and Hold tasks were compared between groups using the technique of wavelet-functional ANOVA (wfANOVA) (32, 33). As we were interested in both the phasic activation patterns and the rest amplitudes before and after each contraction, we selected time windows that included 3 sec before and after each contraction. Using Subject as a random effect, all task repetitions from each subject were included in the wfANOVA model. For each task, the RMS EMG waveforms were transformed into the wavelet domain, allowing temporally localized features to be represented by a small number of orthogonal (independent) wavelet coefficients. These coefficients were then statistically tested between groups using a one-way ANOVA at each time point to evaluate if there were differences in PFM activation patterns between groups. Significant *between-group* contrasts were identified and transformed back from the wavelet domain into the time domain for visualization.

### Sample size estimation

Sample size calculation was performed *a priori* using G*Power. Calculations were performed considering a repeated measures design, a power of 0.80, a probability of error α 0.05, and an effect size of 0.25 calculated by the partial n² of 0.06. According to the study design, it was considered for the calculation two groups (GDM and non-GDM) and three measurements (i.e., three time points), an estimated correlation among repetition measures of 0.5, and non-sphericity correction of 1; the estimated sample size required was at least 28 participants (14 in each group).

### Statistical methods

The software IBM SPSS Statistics for Windows, version 20.0 (IBM Corp., Armonk, N.Y., USA), was used for statistical analysis. The chi-square test or Fisher’s exact test was applied to compare the nominal data between groups. The Mann–Whitney U test was applied to compare independent categories on table. The EMG parameters were tested using a two-way general linear model (GLM), with Group (GDM, non-GDM) and Time Point (1–3) as factors, with repeated measures on the time-point factor (i.e., within-subject). The hypothesis of sphericity was tested by the Mauchly test, and when the sphericity was rejected, the Greenhouse–Geisser correction was applied. When a significant main effect or interaction effect was found, pair-wise *post-hoc* tests were applied using Bonferroni correction and relative percentages were used to show the magnitude of differences on the statistical tests. Furthermore, as mentioned previously, the full RMS EMG waveforms from the Flick and Hold tasks were compared between groups using the technique of wfANOVA. Differences were considered statistically significant if p <.05.

## Results

### Flow of participants through the study

**Figure 3:**
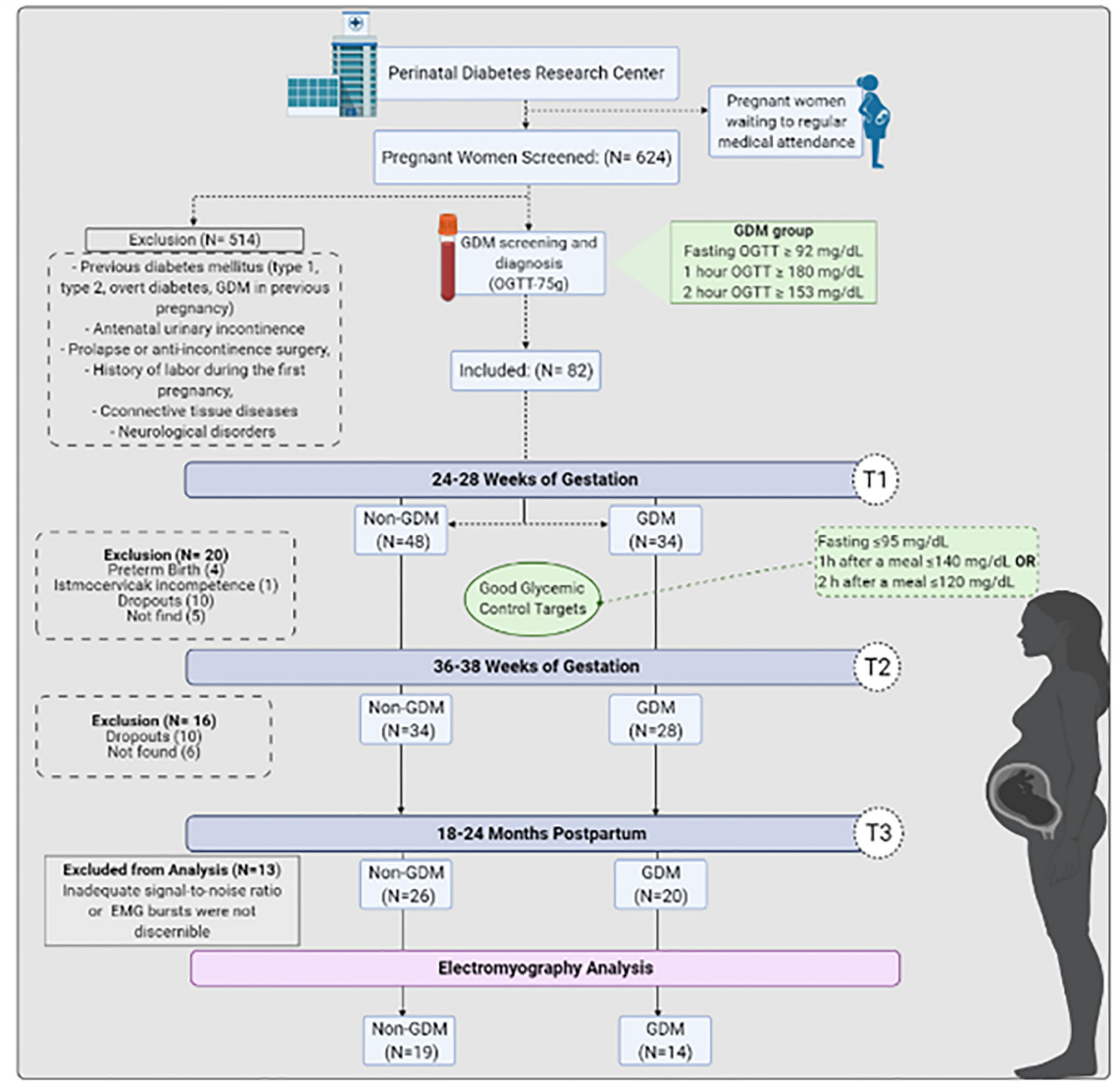
GDM women’s screening, diagnosis, enrollment, follow-up analysis and reasons for signal exclusion from analysis.

The flowchart in **Figure 3** illustrates the number of women examined at each time point and the reasons for dropout. Among all initially included participants (n = 82), 48 women were allocated in the non-GDM group and 34 in the GDM group. Sixty-two participants remained on T2 (34 non-GDM and 28 GDM), and 46 returned to complete T3 on postpartum (26 non-GDM and 20 GDM). The reasons for dropout were not related with DMG complications. Due to technical failure related to an inappropriate signal-to-noise ratio (maybe attributed to probe movement, inherent equipment/ambient noise) and/or not detectable EMG burst (maybe due to intrinsic reasons), the inclusion of 13 participants was unfeasible. Therefore, the EMG analyses were proceeded with participants who had all time points completed and with good EMG signal quality (19 non-GDM and 14 GDM).

No significant group differences were found in participant characteristics during gestation or postpartum (**Table 1**). The glucose tolerance test values, as expected, showed marked group differences on fasting, 1 and 2 h after OGTT.

**Table 1:** Average participant characteristics for non-GDM and GDM groups along time points.

Variable		Non-GDM (n = 19)	GDM (n = 14)	p
Ethnicity	Caucasian	13 (68.4%)	7 (50%)	.472^$^
	Other	6 (31.6%)	7 (50%)	
Smoking in pregnancy		0 (0.0)	0 (0.0)	1.000^#^
Smoking postpartum		0 (0.0)	0 (0.0)	1.000^#^
Education level–min. high school		7 (36.8%)	4 (28.6%)	.453^$^
Diabetes postpartum		0 (0.0)	0 (0.0)	1.000^#^
Age (years)^1^		26 (18-39)	29 (18-40)	.529^$^
BMI (kg/m^2^) pre-pregnancy		23.6 (19.1-30.7)	25.2 (18.5-34.7)	.900*
BMI (kg/m^2^) at 24–30 weeks		26.4 (19.1-32.9)	25.9 (21.6-37.4)	.843*
BMI (kg/m^2^) at 36–38 weeks		28.4 (21.2-34.0)	27.7 (22.8-38.7)	.928*
BMI (kg/m^2^) postpartum^3^		24.6 (17.1-35.2)	24.2 (18.3-36.6)	.957*
Weeks of gestational^1^		26.0 (24.2-29.0)	27.0 (24.0-29.0)	.506*
Weeks of gestational^2^		36.0 (35.3-38.0)	36.0 (35.0-38.0)	.843*
Postpartum time		24.0 (18.1-24.0)	19.5 (18.0-24.0)	.123*
Delivery mode	C-Section	14 (73,3%)	11 (78,6%)	.746^$^
	Vaginal	5 (26,3%)	3 (21,4%)	
Newborn weight at birth (grams)		3100 (2205-4100)	3150 (2560-3935)	.577*
Blood glucose (mg/dL) ^1^		84 (65-90)	88 (76-98)	.077*
OGTT (mg/dL)—fasting^1^		76.0 (71.7-90.0)	92.0 (76.0-124.0)	.000*
OGTT—1 h (mg/dL) ^1^		122.0 (76.7-163.0)	152.0 (82.0-211.0)	.012*
OGTT—2 h (mg/dL) ^1^		110.0 (64.6-148.0)	138.5 (72.0-179.0)	.019*

Non-GDM, non-gestational diabetes mellitus group; GDM, gestational diabetes mellitus group; BMI, body mass index; OGTT, oral glucose tolerance test. ^1^Evaluation at 24–30 weeks of gestation. ^2^Evaluation at 36–38 weeks of gestation. ^3^Evaluation at 18–24 months postpartum. Data are presented in median (minimum–maximum) or absolute frequency (n) and percentage (%). p-values are based on *Mann–Whitney U, ^$^chi-square, and ^#^Fisher’s exact. Significance p < 0.05. p-values represent the results from the relevant statistical tests.

**Table 2** shows the average (and standard deviation) of the parameters extracted from the RMS EMG divided across groups and time points, as well as the results from the GLM. The variables evaluated during the 60-sec pre-baseline did not differ between or within groups. During the 1-sec Flick contractions, there was an interaction between time points and groups on the average EMG amplitude [F(1.619,43.759) = 4.568; p = .022]. *Post-hoc* analyses revealed that the GDM group decreased (-11.0%) the activation levels from T1 to T3 (p = .040). Additionally, during T1, the GDM group showed a higher (+12.1%) slope after onset (increased rate of EMG activity) compared to the non-GDM group [main effect of group, F (1,27) = 4.504; p = .043, *post-hoc* p = .043]. Moreover, the main effects of time point revealed that, independent of group, women took more time to reach peak EMG, during T1 compared to T3 (non-GDM: 22.2% and GDM: +11.1%) [F (2,54) = 8.354; p <.001, *post-hoc* p <.001]; the task duration was lower at T1 compared to T2 (non-GDM: -16.6% and GDM: -5.5%), and T3 (non-GDM and GDM: -16.6%) in both groups decreased from T1 to T2 [F (2,54) = 9.536; p <.001, *post-hoc* T1 to T2 p = .008 and T1 to T3 p <.001]; and the rate of EMG increase after onset was lower during T1 compared to T2 (non-GDM: +48.5% and GDM: +6.6%) [F (2,54) = 3.633; p = .033, *post-hoc* p = .041]. Finally, interactions between Group and Time Points revealed that the standard deviation of the EMG amplitude on the non-GDM group increased from T1 to T2 (+12.1%) [F (2,54) = 3.345; p = .043, *post-hoc* p = .031] and the EMG slope before offset (decrease rate of EMG activity) was less intense at T1 compared to T2 (-55.4%) and T3 (-47.4%) in the non-GDM group [F (2,54) = 4.812; p = .012 *post-hoc* T1 to T2 p = .005 and T1 to T3 p = .015].

**Table 2:** Group mean ± standard deviation (across subjects) of the parameters extracted from the EMG signals at each task of the Glazer protocol.

	Non-GDM (19)			GDM (14)			General linear model		
EMG variables	T1	T2	T3	T1	T2	T3	p	p	p
	time point	time point	time point	time point	time point	time point	between groups	Interaction group *vs*. time points	Time points
**60-sec pre-baseline (rest)**									
Average (%)	8.5 ± 8.0	7.7 ± 5.0	8.3 ± 6.2	6.2 ± 3.9	5.8 ± 3.2	8.3 ± 3.1	.461	.760	.664
Peak (%)	21.9 ± 19.4	16.8 ± 10.2	18.0 ± 11.8	17.1 ± 11.7	13.9 ± 6.1	24.4 ± 8.8	.907	.411	.415
Amplitude SD (%)	2.5 ± 1.8	2.3 ± 1.4	2.3 ± 1.4	2.1 ± 1.4	1.7 ± 0.6	3.4 ± 1.4	.951	.258	.264
Amplitude CV (%)	32.7 ± 6.2	30.0 ± 8.7	30.3 ± 6.8	33.6 ± 6.3	32.6 ± 10.3	42.0 ± 14.9	.096	.199	.310
Task duration	57.1 ± 4.9	59.7 ± 0.6	59.4 ± 0.5	58.6 ± 2.2	59.4 ± 0.4	58.5 ± 0.4	.854	.347	.201
SNR	1.5 ± 1.0	1.3 ± 0.6	1.3 ± 0.4	1.2 ± 0.4	1.5 ± 0.6	1.2 ± 0.2	.833	.466	.889
**1-sec phasic (Flicks)**									
Average (%)	50.0 ± 9.0	52.2 ± 4.7	51.9 ± 5.9	55.2 ± 4.7^a^	52.7 ± 5.3	49.1 ± 5.2^a^	.558	**.022**	.233
Peak (%)	83.6 ± 11.1	87.9 ± 5.6	86.8 ± 6.4	89.2 ± 5.0	86.9 ± 6.1	84.0 ± 5.7	.711	.063	.495
Amplitude SD (%)	20.5 ± 3.2^b^	23.0 ± 3.0^b^	22.2 ± 3.3	22.6 ± 2.4	22.1 ± 3.0	21.7 ± 2.9	.806	**.043**	.287
Amplitude CV (%)	42.1 ± 8.3	44.7 ± 7.0	43.6 ± 7.0	41.6 ± 6.3	42.8 ± 7.6	44.7 ± 7.3	.825	.626	.290
Time from onset to peak (sec)	0.9 ± 0.2^&^	0.8 ± 0.2	0.8 ± 0.2^&^	0.9 ± 0.3*	0.8 ± 0.3	0.7 ± 0.2*	.978	.146	**<.001**
Time from peak to offset (sec)	1.0 ± 0.3	0.7 ± 0.2	0.8 ± 0.3	0.9 ± 0.3	0.8 ± 0.3	0.9 ± 0.2	.442	.172	.052
Task duration	1.8 ± 0.4^&$^	1.5 ± 0.3^&^	1.5 ± 0.5^$^	1.8 ± 0.4*^£^	1.7 ± 0.3*	1.5 ± 0.2^£^	.650	.306	**<.001**
Slope after onset (%/sec)	111.7 ± 30.8^c&^	165.7 ± 52.3^&^	152.9 ± 55.5	174.5 ± 62.5c*	186.1 ± 64.2*	169.3 ± 67.9	**.043**	.117	**.033**
Slope before offset (%/sec)	-96.3 ± 64.6^de^	-149.7 ± 61.9^d^	-142.8 ± 63.4^e^	-130.5 ± 69.4	-124.7 ± 53.6	-115.4 ± 43.2	.741	**.012**	.110
Slope before peak (%/sec)	100.9 ± 57.1	125.3 ± 55.3	131.0 ± 38.1	118.2 ± 50.7	116.6 ± 67.0	120.8 ± 38.1	.971	.423	.367
Slope after peak (%/sec)	-116.8 ± 46.9	-148.4 ± 71.1	-114.9 ± 42.0	-99.5 ± 32.8	-116.1 ± 41.1	-129.0 ± 38.3	.254	.174	.162
SNR	21.1 ± 15.1	24.6 ± 13.6	28.4 ± 36.3	22.8 ± 16.8	30.9 ± 22.0	14.8 ± 10.8	.701	.157	.437
**10-sec hold**									
Average (%)	52.2 ± 15.5	56.4 ± 20.0	52.9 ± 16.6	48.0 ± 10.6	51.4 ± 17.3	46.7 ± 18.3	.241	.962	.437
Peak (%)	101.2 ± 27.9	106.3 ± 32.3	99.7 ± 21.5	95.2 ± 10.8	98.2 ± 18.6	95.9 ± 29.2	.268	.941	.729
Amplitude SD (%)	17.8 ± 5.6	19.0 ± 5.7	17.5 ± 4.5	17.6 ± 3.0	18.1 ± 3.4	16.4 ± 5.4	.491	.916	.383
Amplitude CV (%)	35.7 ± 9.1	35.6 ± 8.3	35.3 ± 9.5	38.1 ± 8.2	38.0 ± 11.0	38.2 ± 14.4	.361	.988	.996
Time from onset to peak (sec)	3.1 ± 1.8	2.4 ± 1.7	2.9 ± 2.0	2.1 ± 1.6	3.2 ± 2.3	2.2 ± 1.8	.548	.019	.715
Time from peak to offset (sec)	7.0 ± 1.8	7.8 ± 1.8	7.4 ± 2.0	8.2 ± 1.5	7.0 ± 2.2^f^	8.4 ± 1.7^f^	.408	**.009**	.313
Task duration	10.1 ± 0.5^&^	10.2 ± 0.4^$^	10.4 ± 0.3^&$^	10.3 ± 0.4*	10.2 ± 0.4^£^	10.5 ± 0.4*^£^	.189	.516	**.026**
Slope after onset (%/sec)	116.9 ± 57.4	144.7 ± 57.0	151.1 ± 50.3	144.1 ± 45.2	160.7 ± 62.4	147.2 ± 71.1	.397	.397	.126
Slope before offset (%/sec)	-81.7 ± 61.0	-83.6 ± 44.9	-97.4 ± 67.1	-60.0 ± 29.4	-80.0 ± 58.8	-75.1 ± 45.1	.266	.626	.356
Slope before peak (%/sec)	121.8 ± 47.3^&^	136.9 ± 59.8	142.2 ± 55.0^&^	125.4 ± 53.9*	124.9 ± 53.2	171.8 ± 76.6*	.588	.310	**.042**
Slope after peak (%/sec)	-129.1 ± 62.6	-123.7 ± 48.4	-130.9 ± 37.8	-105.1 ± 32.5	-107.6 ± 43.1	-123.9 ± 75.5	.190	.781	.577
SNR	25.8 ± 22.3	23.7 ± 17.4	26.1 ± 26.8	17.0 ± 13.2	23.8 ± 14.0	9.7 ± 4.4	.053	.186	.414
**60-sec endurance**									
Average (%)	38.7 ± 14.5	48.7 ± 28.3	39.9 ± 14.1	38.4 ± 11.7	37.3 ± 10.2	40.2 ± 36.5	.540	.602	.790
Peak (%)	116.8 ± 50.0	114.9 ± 58.6	109.1 ± 12.8	92.7 ± 16.7	93.4 ± 22.4	111.8 ± 104.5	.337	.673	.915
Amplitude SD (%)	17.3 ± 6.6	18.4 ± 10.8	18.3 ± 4.5	13.4 ± 4.7	13.4 ± 3.1	14.8 ± 13.5	.091	.943	.894
Amplitude CV (%)	46.8 ± 13.9	38.8 ± 10.5	51.3 ± 20.5	36.1 ± 11.6	37.0 ± 9.2	42.8 ± 21.9	.249	.391	.030
Task duration	60.9 ± 1.0	60.4 ± 1.0	60.3 ± 1.4	59.5 ± 2.4	60.7 ± 1.6	59.2 ± 3.0	.174	.244	.333
SNR	14.0 ± 12.0	17.9 ± 12.7	13.0 ± 10.2	10.7 ± 6.5	16.4 ± 7.2	7.2 ± 4.4	.275	.698	.067
**60-sec post-baseline (rest)**									
Average (%)	10.0 ± 7.7	9.0 ± 3.3	8.4 ± 2.4	7.0 ± 0.6	3.5 ± 0.9	10.4 ± 4.5	.234	.137	.235
Peak (%)	25.0 ± 12.3	22.6 ± 7.8^&^	24.2 ± 12.5^&^	17.5 ± 3.7	9.1 ± 2.1*	33.2 ± 14.3*	.304	.054	**.031**
Amplitude SD (%)	3.4 ± 1.5	3.2 ± 1.0^f^	3.3 ± 1.7	2.4 ± 0.4	1.2 ± 0.1^f^	3.6 ± 1.4	**.044**	.176	.138
Amplitude CV (%)	46.8 ± 33.6	37.2 ± 10.1	37.9 ± 11.1	33.8 ± 6.2	34.8 ± 6.2	36.7 ± 11.2	.322	.753	.782
Task duration	56.2 ± 3.6	56.9 ± 1.6	57.9 ± 0.8	55.7 ± 0.3	57.3 ± 1.9	55.1 ± 1.7	.158	.269	.532
SNR	2.0 ± 1.4	1.4 ± 0.6	1.5 ± 0.9	1.3 ± 0.4	1.1 ± 0.2	1.4 ± 0.4	.273	.729	.622

Non-GDM, non-gestational diabetes mellitus group; GDM, gestational diabetes mellitus group; SD, standard deviation; CV, coefficient of variation; %, percentage; Sec, seconds. Same letters and symbols indicate differences detected by post-hoc (Bonferroni) contrasts test; p value < 0.05.

Results are presented from the two-way general linear model (GLM) using factors Group (non-GDM, GDM) and Time Point (T1: 24–30 weeks of gestation, T2: 36–38 weeks of gestation, T3: 18–24 months postpartum) as factors, with repeated measures on Time Point.

During the 10-sec hold task, there was an interaction between Group and Time Point on the time from peak to EMG offset [F (2,62) = 5.068; p = .009], indicating that the GDM group took less time to return to baseline after the peak in T3 compared to T2 (-20%) (p = .023). The main effects of Time Point revealed that, independent of group, task duration was larger at T3 than both T1 (non-GDM: +2.8% and GDM: +1.9%) and T2 (non-GDM: +1.9% and GDM: +2.8%) [F(1.580,46.735) = 3.895; p = .026, *post-hoc* T1 to T3 p = .023 and T1 to T3 p = .023] and that the EMG slope before peak was greater at T3 compared to T1 (non-GDM: +16.7% and GDM: +37%) [F (2,62) = 3.335; p = .042, *post-hoc* p = .035].

During the last PFM contraction task of the protocol, the 60-sec hold, there were no significant main effects of interactions with Group or Time Point. During the 60-sec post-baseline rest period, there was a significant effect of Group on the standard deviation of EMG amplitude, and the GDM group showed a lower amplitude (-62.5%) compared to the non-GDM group [F (1,10) = 5.319; p = .044]. A main effect of Time Point revealed that the peak amplitude was greater at T3 than at T2 (non-GDM: +7% and GDM: +264.8%) [F(2,20) = 4.152; p = .031, p = .023] independent of group.

**Figure 4:**
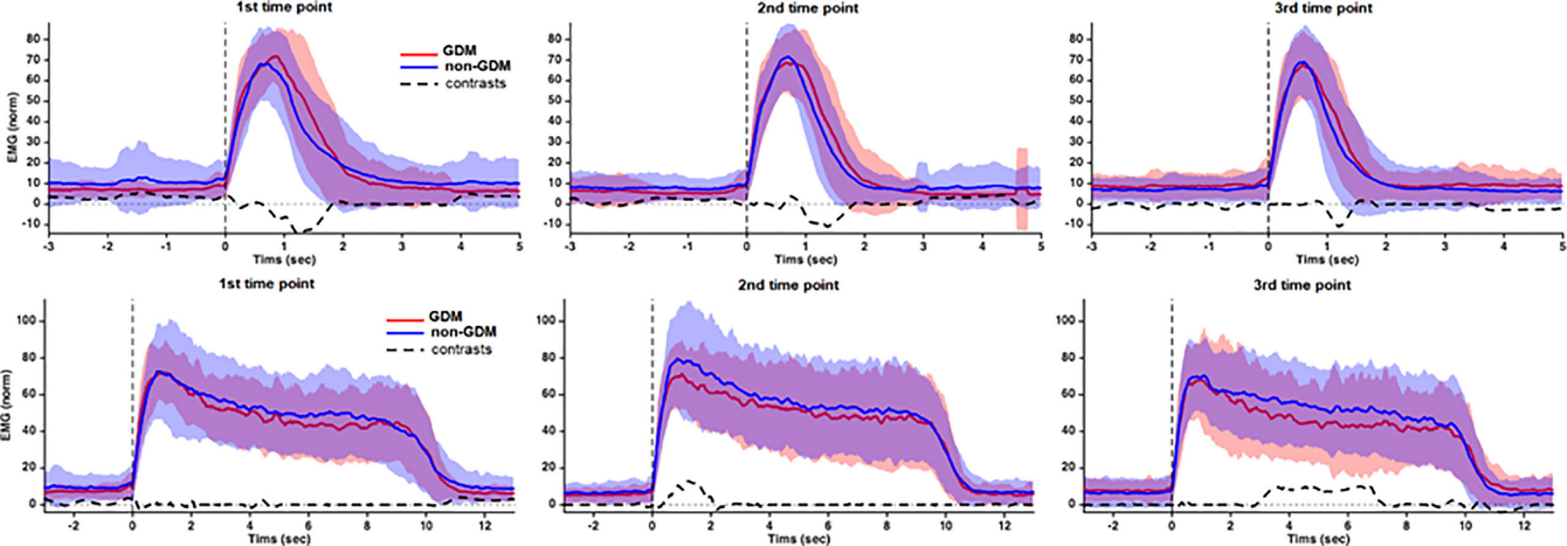
Group average and SD of the RMS EMG during the 1-sec Flick and 10-sec Hold PFM contraction tasks from Glazer protocol. Before averaging, the EMG patterns from each subject was expressed as percentage of the peak recorded during the 1-sec Flick contractions. Positive contrasts indicate that GDM < non-GDM. Source: Diamater Study Group.

**Figure 4** shows the results of the wfANOVA analysis, with the average EMG patterns of each group and the significant Group contrasts during the Flick and Hold PFM contraction tasks at each time point. The significant contrasts indicate that, during the Flick contractions, the GDM group generally had a greater PFM EMG amplitude than non-GDM after ~1 sec of contraction, suggesting to return from peak amplitude to baseline level contractions. During the 10-sec Hold contractions, the non-GDM group activated the PFM at higher contraction intensities than the GDM group at both time points T2 and T3, although the timing of the contrasts differed between time points: At T2, the GDM group had a lower initial peak amplitude during Hold but similar amplitudes after ~2 sec of contraction; at T3, the initial peaks from both groups had similar (normalized) amplitudes, after which the levels of PFM activation decreased faster for the GDM group, remaining lower than for the non-GDM group until near the end of the contraction.

## Discussion

This study assessed PFM EMG patterns from pregnancy to long-term postpartum (18–24 months) in women with and without GDM. Using a well-established protocol for pelvic floor assessment, we reproduced a similar sequence of PFM contractions requested in clinical consultations, commonly used to identify the motor strategy during brief and sustained PFM tasks. No significant group differences were found during the Baseline-pre and Endurance tasks, and only minor differences during Baseline-post. During 1-sec Flick contractions, the EMG activation of all participants decreased on postpartum compared to T1. Wavelet analysis showed that, although the GDM group achieved peak PFM EMG amplitudes similar to the non-GDM, they took longer to return to baseline levels. During 10-sec Hold contractions, the GDM group sustained lower levels of PFM activation than the non-GDM group at both T2 and T3.

Our study was based on evidence of changes in physiological and anatomical factors in the female PFM demonstrated by morphological studies in pregnant rats and humans (6). A reduced ratio of fast to slow fibers and a co-localization of fast and slow fibers have been observed in striated urethral muscle of diabetic pregnant rats compared with non-diabetics and non-pregnant rats (8, 9). More recently, similar findings were found in rectus abdominis muscles of pregnant women with GDM, who showed a decreased cross-sectional area of both slow and fast muscle fibers, in addition to a decreased number of fast fibers and an increased number of slow fibers (6, 34).

It is reasonable that morphologic and metabolic changes in PFMs are likely to contribute to UI (30, 35, 36). Indeed, higher UI prevalence and severity have been associated with hyperglycemic disturbances not only during pregnancy (2, 3, 37) but also on prediabetes and clinical diabetes (38, 39). Three-dimensional ultrasonography during rest showed that there is a decrement of the thickness of the levator ani muscle (17) during pregnancy, which is consistent with previous morphological findings of a myopathic process on musculoskeletal tissue of GDM pregnant women (6, 15). However, conclusive evidence to support this relationship has not yet been assessed due to the lack of studies assessing pelvic floor function by direct measures (40–42), particularly on pregnancy until medium and long-term postpartum (13).

Autonomic neuropathic dysfunctions in the bladder are associated with hyperglycemia (43, 44). Besides it, findings on external anal sphincter using electrophysiological methods showed that diabetic polyneuropathy caused by clinical diabetes mellitus (DM) affects the pudendal nerve by an increase in motor unit action potentials (MUPs), mean duration, mean amplitude, mean phases, satellite rate, and percentage of long-duration MUPs and polyphasic potentials (40, 45).

Previous studies in pregnant woman populations with GDM showed differences in PFM activation between GDM and non-GDM groups at 24–30 and 36–38 weeks of gestation, particularly during rest and hold contractions (16). Although different EMG processing and normalization methods hinder direct comparison with the present study, our results complement previous findings by demonstrating that, as they approach the end of pregnancy, women with GDM show reduced ability to perform brief PFM contractions and to sustain long PFM contractions at the same level as their non-GDM counterparts.

Motor control studies have shown that the reduction in EMG amplitude secondary to muscle weakness could not fully explain the UI, and they showed that the pre-activation of the PFM could have a great contribution on the continence mechanisms (30). Other mechanisms should be addressed to explain better the motor strategy used by the GDM group along (46). Thus, we decided to include, besides the amplitude and peak quantification; analyses of contraction oscillation; temporal analyses related to onset, peak, and offset; and rate of recruitment during the begin and end of the contraction and the peak.

The findings from the 1-sec Flick contractions showed that the GDM group decreased their levels of PFM activation from T1 to T3, whereas the non-GDM group maintained similar levels of activation along time points. We believe that the significant increase in amplitude standard deviation clinically implies about amplitude variability during the same task the non-GDM group could contribute to allow the non-GDM group to maintain the level of activation. The implications of low or high variability is still controversial in literature, but there is evidence that a higher variability may represent an adaptive mechanism to maintain the task performance (47).

The impairments in PFM function observed in women with GDM have been attributed to physiological and anatomical changes to the musculoskeletal system, namely, reduced cross-sectional area and reduced number of fast fiber type, in addition to impairments in ionic channels, as well as fat infiltration and proliferation of connective tissue in the PFM (48). Nevertheless, we cannot exclude other confounding factors, including the volitional component (i.e., choose not to activate) and technical aspects inherent to EMG acquisition which could affect both groups (49).

Both groups achieved peak quicker on T3 compared to T1 on the flicks task. As this characteristic was the same on the groups and no differences were found between T1 and T2, the pregnancy itself may have an implication on it. A quicker response of the pelvic floor is important mainly when intra-abdominal pressure is higher to promote continence. Other studies should consider exploring the latency of PFM onset to peak in comparing it with other structures involved on the modulation of intra-abdominal pressure (50).

Although our protocol had a standard task duration, we observed that both groups decreased duration in the flicks contractions on T2 and T3 compared to T1 to T2 in both groups and achieved peak on T3 quicker than T1. We believe that this is probably a result of a learning effect: as participants got familiar with the tasks, both groups were able to reach peak amplitude more quickly than before, increasing the rate of EMG activity (slope after onset). Additionally, we expected on T1 that the groups may have the same recruitment characteristics, but the GDM group activates PFM around 60% more per second compared to non-GDM.

Concerning the deactivation on the end of the task (slope before offset), the non-GDM decreased the rate of EMG activity intensely from T1 to T2 and from T1 to T3. The GDM group used the same strategy to relax pelvic floor muscle along time points. When comparing the full RMS EMG waveforms between groups, we found that, at all three time points, the GDM group took longer T1 to return from peak amplitude to baseline levels, as revealed by a higher EMG amplitude compared to non-GDM after peak EMG. This observation wave characteristic is corroborated by a recent study applying the same protocol to continent and incontinent women that found that the incontinent group took more time to relax after Flick contractions (51).

On the 10-sec Hold tasks, whereas traditional amplitude measurements were not able to identify major differences between groups or time points, the analyses in the wavelet domain found a reduced EMG amplitude in the GDM group compared to non-GDM at time points T2 and T3, which means that when events along the task are taken into consideration, different motor control patterns are found between groups along the task duration in each time point. During T1, the motor pattern was mostly similar between groups. It could be explained by the fact that this is the screening period to GDM, so it is the point that glycemia starts to get higher and maybe there is no drastic influence on muscle yet. Also, the discrete but significant differences on T2 could be explained by the fact that the cross-section area of slow fibers are decreased in the GDM group (6, 15). Although the capacity of the morphological recovery on postpartum is unknown, our findings suggest that PFM control continues to be impaired postpartum in the GDM group. Additionally, the GDM group took more time to return from peak to offset from T2 and T3. Although the task duration statistically increased from T1 to T2 and T3, it was less than 1 sec and may not be relevant clinically.

During post-baseline resting, there were differences related to the peak from T2 to T3 in both groups and the GDM group on T2 oscillated less during the final resting. Although significant, these two characteristics without an additional change on average amplitude, clinically, do not provide a valuable reflection about the task in general.

EMG is a valuable but challenging method to evaluate PFM function; hence, interpretation of the present results should be made with caution to avoid mistaken conclusions (52). Although the findings from the present study may be partially explained by morpho-pathological processes involved in GDM, there are several concerns to consider: first, the test–retest reliability of PFM EMG amplitude along time points shows heterogeneity among studies in the literature (53, 54). Previous studies have suggested that this heterogeneity arises mainly due to electrode movement, which contaminates the signal with motion artifact and changes the population of motor units recorded, making it difficult to evaluate the same motor units across different time points (54). In addition, some studies have assessed raw EMG amplitudes, which turns the external validity and results comparisons unfeasible.

This novel cohort study evaluated PFM activity in pregnant women with and without GDM at three distinct time points during and after delivery. We argue that the strengths of the study were that (i) we only included continent pregnant women; (ii) we excluded from analysis participants who did not complete the cohort entirely; (iii) only high-quality EMG was included on the analysis, confirmed by high SNR and absence of signal artifacts; (iv) we assessed many different parameters, including traditional amplitude and timing parameters and the assessment of the full RMS EMG waveform, in an attempt to perform a comprehensive assessment of the motor strategies during PFM contractions; and (v) the EMG amplitude of each subject was normalized by the maximal voluntary activation to allow comparisons between groups and time points.

Nevertheless, we also acknowledge some limitations in our study, which should be taken into consideration in future studies. First, we had a relatively high dropout rate, which is a common problem in cohort studies and randomized controlled trials assessing pregnant women (22), probably underpinned by the major changes in women’s life that accompany pregnancy and delivery. Second, the assessment of vaginal pressure or force, concomitant with EMG, would have been valuable to assess changes in force-generating capacity and allow more reliable estimates of maximal voluntary contractions (30). In addition, we also did not consider fatigue measurements, mainly because as shown by other authors we have a gap on literature about standardized protocols to assess PFM fatigue (55). Third is the employment of intravaginal high-density surface electromyography to allow others such as the number of motor unit action potentials by the decomposed signal (56). Finally, the use of vaginal probes with suction, designed to minimize movement artifacts and ensure optimal electrode alignment with the muscle fiber direction, is likely to enhance the technical quality of the EMG recordings.

## Conclusion

Our findings show impaired PFM motor control strategies on pregnant women with GDM compared to non-GDM during execution of 1-sec Flick and 10-sec Hold contractions during pregnancy and 18–24 months postpartum. Taken together, these results suggest that differences on motor behavior of GDM women arise in late pregnancy and exacerbate on postpartum.

## Research implications

To the best of our knowledge, this is the first study to provide information about PFM neuromuscular strategy of woman GDM in a long-term follow-up. Further studies should be necessary to investigate the influence of this strategy on PFM strength and pelvic floor dysfunctions. This additional information should be important to delineate preventive and therapeutic strategies on this population.

## Data availability statement

The authors confirm that all data underlying the findings are fully available without restriction. All relevant data are within the paper. Access to the data should be required to the corresponding author.

## Ethics statement

This study was reviewed and approved by Institutional Ethical Committee of Botucatu Medical School of Sao Paulo State University. The patients/participants provided their written informed consent to participate in this study.

## Author contributions

CBP and SKN are first authors on this work. MVCR and AMPB are last authors on this work. These authors contributed equally to this work. CBP, SKN, FAP, CISF, CBP, AMPB, MVCR e Diamater Study Group contributed to conception and design of the study. CBP, SKN, FAP, CISF e Diamater Study Group were responsible for participants during the study. CBP, GTAN, SES organized the database. CBP e SES performed EMG analysis and statistical analysis CBP wrote the first draft of the manuscript. AMPB, MVCR, CRP, SES reviewed the first draft. All authors contributed to manuscript revision, read, and approved the submitted version.

## Funding

The authors have no potential conflicts of interest related to this study. Funding received: Supported by Sao Paulo Research Foundation protocol number 2016/01743-5 and 2021/10665-6. Brazilian Federal Agency for Support and Evaluation of graduate Education/SD (Coordenação de Aperfeiçoamento de Pessoal de Nível Superior, CAPES/DS). The funders had no role in study design, data collection and analysis, decision to publish, or preparation of the manuscript.

## Acknowledgments

We would like to give our great appreciation to the pregnant women who participated in this study.

## Conflict of interest

The authors declare that the research was conducted in the absence of any commercial or financial relationships that could be construed as a potential conflict of interest.

## Publisher’s note

All claims expressed in this article are solely those of the authors and do not necessarily represent those of their affiliated organizations, or those of the publisher, the editors and the reviewers. Any product that may be evaluated in this article, or claim that may be made by its manufacturer, is not guaranteed or endorsed by the publisher.

## Diamater study group

Rudge MVC, Barbosa AMP, Calderon IMP, Souza FP, Berghmans B, Thabane L, Junginger B, Graeff CFO, Magalhães CG, Costa RA, Lima SAM, Kron-Rodrigues MR, Felisbino S, Barbosa W, Campos FJ, Bossolan G, Corrente JE, Nunes HRC Abbade J, Rossignoli PS, Pedroni CR, Atallah AN, Di Bella ZIKJ, Uchoa SMM, Hungaro MA, Mareco EA, Sakalem ME, Martinho N, Hallur LSR, Reyes DRA, Alves FCB, Marcondes JPC, Prudencio CB, Pinheiro FA, Sartorão Filho CI, Quiroz SBCV, Pascon T, Nunes SK, Catinelli BB, Reis FVDS, Oliveira RG, Barneze S, Enriquez EMA, Takano L, Carr AM, Magyori ABM, Iamundo LF, Carvalho CNF, Jacomin M, Avramidis RE, Silva AJB, Orlandi MIG, Dangió TD, Bassin HCM, Melo JVF, Takemoto MLS, Menezes MD, Caldeirão TD, Santos NJ, Lourenço IO, Marostica de Sá J, Caruso IP, Rasmussen LT, Garcia GA, Nava GTA, Pascon C, Bussaneli DG, Nogueira VKC, Rudge CVC, Piculo F, Prata GM, Barbosa VP.
